# Pulmonary blood density quantified by CMR is reduced in newly diagnosed systemic sclerosis, consistent with pulmonary arteriolar proliferation

**DOI:** 10.1186/1532-429X-13-S1-P387

**Published:** 2011-02-02

**Authors:** Mikael Kanski, Hakan Arheden, Dirk M Wuttge, Gracijela Bozovic, Roger Hesselstrand, Martin Ugander

**Affiliations:** 1 Skåne University Hospital and Lund University, Lund, Sweden

## Introduction

Systemic sclerosis (SSc) is associated with an increased risk of developing pulmonary fibrosis or pulmonary arterial hypertension. Pulmonary arteriolar proliferation in SSc might yield a reduction in the pulmonary blood density (PBD). However, we hypothesized that PBD would be increased in SSc due to increased pulmonary artery pressure and distension.

## Purpose

We aimed to use cardiovascular magnetic resonance imaging (CMR) to explore the pulmonary blood volume (PBV), the PBV variation over the cardiac cycle (PBVV), and the PBD as possible measures of pulmonary involvement in newly diagnosed SSc.

## Methods

Twenty-seven SSc patients (9 men, 30-79 years, 18 limited cutaneous SSc, 3 diffuse cutaneous SSc, and 6 early SSc) and 20 healthy subjects (13 men, 18-46 years) underwent CMR. The PBV was calculated as the product of cardiac output determined by velocity encoded CMR, and the pulmonary transit time determined as the time for a 2 ml intravenously administered contrast bolus to pass from the pulmonary trunk to the left atrium, as previously validated. The lung volume was determined by planimetry using transversal MR images covering the lungs. The PBD was defined as the PBV divided by the lung volume. Also, the blood flow in the pulmonary artery and the pulmonary veins was measured using velocity encoded CMR. The PBVV was calculated by integration of the difference in arterial and venous pulmonary flow over the cardiac cycle.

## Results

Compared to healthy subjects, SSc patients had lower PBV (460±85 vs 602±125ml, p<0.01), lower PBD (16±5 vs 21±2%, p<0.001; 15/27 (56%) had PBD below normal limits), but no difference in PBVV/stroke volume (40±8 vs 46±10%, p=0.12). PBD correlated with Doppler echocardiography estimated pulmonary artery pressure (r=-0.36, p<0.05) and the diffusion capacity for carbon monoxide (DLCO) in the lungs (r=0.44, p=0.02), but was not affected by pulmonary fibrosis by high-resolution computed tomography (p=0.34).

## Conclusions

Compared to healthy subjects, newly diagnosed SSc patients have a reduced amount of blood in the pulmonary vasculature (PBD), and PBD is inversely related to pulmonary artery pressure. The findings are consistent with an involvement of pulmonary arterioles in SSc. Also, this is the first human study to quantify the pulmonary blood density by CMR. Further studies are justified to assess the utility of PBD as a diagnostic and prognostic measure of pathophysiological changes in cardiopulmonary disease.

